# Evaluation of Messenger RNA From COVID-19 BTN162b2 and mRNA-1273 Vaccines in Human Milk

**DOI:** 10.1001/jamapediatrics.2021.1929

**Published:** 2021-07-06

**Authors:** Yarden Golan, Mary Prahl, Arianna Cassidy, Christine Y. Lin, Nadav Ahituv, Valerie J. Flaherman, Stephanie L. Gaw

**Affiliations:** 1 Department of Bioengineering and Therapeutic Sciences, University of California, San Francisco; 2 Institute for Human Genetics, University of California, San Francisco; 3 Department of Pediatrics, University of California, San Francisco; 4 Division of Maternal-Fetal Medicine, Department of Obstetrics, Gynecology, and Reproductive Sciences, University of California, San Francisco

## Abstract

This cohort study analyzes milk samples from lactating mothers to determine if COVID-19 vaccine–related messenger RNA was detectable in human milk after vaccination.

Messenger RNA (mRNA) vaccines against COVID-19 were recently approved under an emergency use authorization.^1^ However, there is a paucity of data regarding vaccine safety in pregnant or lactating individuals who were excluded from phase 3 clinical trials,^2,3^ and many mothers have declined vaccination or decided to discontinue breastfeeding (temporarily or permanently) due to concern that maternal vaccination may alter human milk. The World Health Organization recommends that breastfeeding individuals be vaccinated and does not advise cessation of breastfeeding following vaccine administration.^4,5^ The Academy of Breastfeeding Medicine states that there is little plausible risk that vaccine nanoparticles or mRNA would enter breast tissue or be transferred to milk,^6^ which could theoretically result in priming of infant immune responses that could alter childhood immunity. However, there are no direct data. To address this knowledge gap, we analyzed milk samples to determine if vaccine-related mRNA was detectable in human milk after vaccination.

## Methods

The institutional review board of the University of California, San Francisco, approved the study. Written informed consent was obtained from all study volunteers in the COVID-19 Vaccine in Pregnancy and Lactation (COVIPAL) cohort study from December 2020 to February 2021. Clinical data were collected by questionnaires. Self-collected milk samples were kept on ice or immediately frozen (at home) until arrival in the laboratory. Samples were collected prior to vaccination and at varied time points up to 48 hours after vaccination. Total RNA was isolated from milk components using the RNeasy Mini Kit (Qiagen). We performed real-time quantitative polymerase chain reaction assay targeting the mRNA used in the COVID-19 mRNA-based vaccines. The BNT162b2 (Pfizer) and mRNA-1273 (Moderna) vaccines were separately inoculated into prevaccination milk samples, which were processed by the same protocols and used as positive controls for this assay (eMethods in the Supplement); prevaccination milk samples were used as negative controls. Based on standard curves, we found that the lower detection limit of our assay was 0.195 pg and 1.5 pg for the BNT162b and mRNA-1273 vaccines, respectively. Because vaccine uptake and mRNA content may differ between milk fractions, we analyzed supernatant and fat separately for all milk samples. Two samples that had sufficient milk cellular material were analyzed separately. A single freeze/thaw cycle of vaccine-inoculated milk samples did not negatively affect mRNA detection compared with fresh samples. Positive controls had higher levels of mRNA-1273 in the fat layer than in the milk supernatant. QuantStudio Software version 1.7.1 (Applied Biosystems) and Prism version 9.1.0 (GraphPad) were used for analyses.

## Results

A total of 7 breastfeeding mothers (mean [SD] age, 37.8 [5.8] years) volunteered for this study (Table). Their children ranged in age from 1 month to 3 years. Postvaccination milk samples were collected 4 to 48 hours after administration of the BNT162b2 (n = 5) or mRNA-1273 (n = 2) vaccines. Analysis of 13 human milk samples collected 24 hours after vaccination, including multiple time points (4 to 48 hours) from a single participant, revealed that none of the samples showed detectable levels of vaccine mRNA in any component of the milk (Figure).

**Table.** Demographic Information on Study Participants and the Samples Collected for Analysis From Each Participant

Participant No.	Age, y	Vaccine type	Time point	Collection method		Milk fraction		
				Fresh	Frozen	Super-natant	Fat	Cells
1	40s	BNT162b2	Prevaccination	NT	T	T	NT	NT
			24 h After dose 1	NT	T	T	T	NT
2	30s	BNT162b2	Prevaccination	NT	T	T	T	NT
			24 h After dose 1	NT	T	T	T	NT
			24 h After dose 2	T	NT	T	T	NT
3	40s	BNT162b2	24 h After dose 1	T	NT	T	T	NT
4	30s	mRNA-1273	8 h After dose 1	NT	T	T	T	NT
			22 h After dose 1	NT	T	T	T	NT
			28 h After dose 1	NT	T	T	T	NT
			33 h After dose 1	NT	T	T	T	NT
			48 h After dose 1	NT	T	T	T	NT
			4 h After dose 2	NT	T	T	T	NT
5	30s	BNT162b2	24 h After dose 1	T	NT	T	T	NT
6	20s	BNT162b2	Prevaccination	NT	T	T	T	T
			24 h After dose 1	T	NT	T	T	T
7	40s	mRNA-1273	Prevaccination	T	NT	T	T	NT
			24 h After dose 2	T	NT	T	T	NT

Abbreviations: NT, not tested; T, tested.

**Figure.**
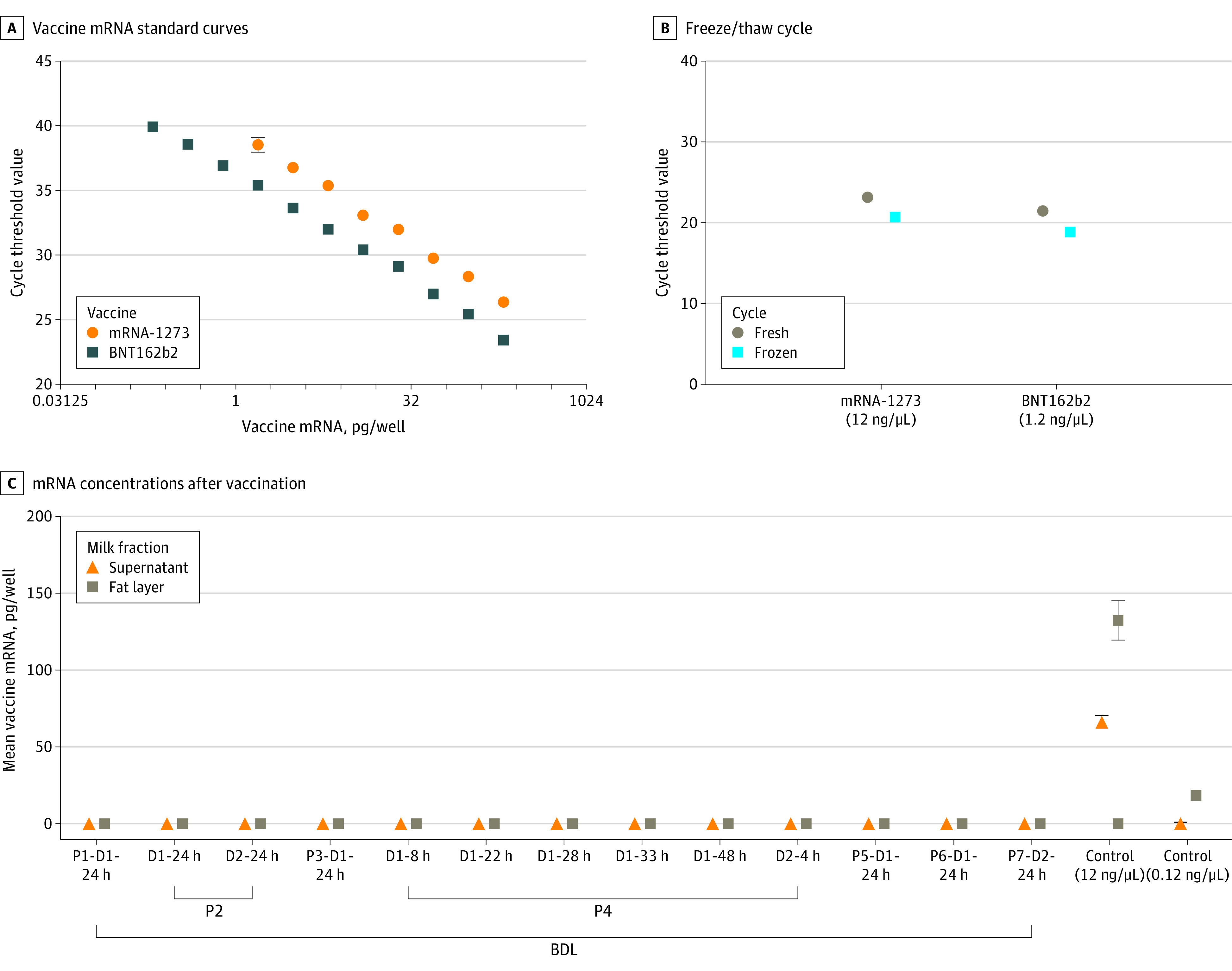
Quantitative Polymerase Chain Reaction Analysis of Human Milk Samples Postvaccination A, Vaccine messenger RNA (mRNA) standard curves. Standard curves for mRNA-1273 (Moderna) and BNT162b2 (Pfizer) vaccines were generated to enable calculation of COVID-19 vaccine mRNA concentration in postvaccination human milk samples (eMethods in the Supplement). B, mRNA-1273 or BNT162b2 vaccines were inoculated into prevaccine milk samples to assess the effect of a single freeze/thaw cycle on the vaccine mRNA detection. C, mRNA concentrations of milk samples 24 hours postvaccine and inoculated controls were calculated based on equations from standard curves. Sample names stand for participant (P) number and if sample was collected after first (D1) or second (D2) vaccine dose, and the number of hours postvaccination. Control samples are the milk samples inoculated with the mRNA-1237 vaccine, with the concentrations of vaccine added to each sample noted. BDL indicates below detectable levels. Error bars indicate SDs.

## Discussion

Vaccine-associated mRNA was not detected in 13 milk samples collected 4 to 48 hours after vaccination from 7 breastfeeding individuals. These results provide important early evidence to strengthen current recommendations that vaccine-related mRNA is not transferred to the infant and that lactating individuals who receive the COVID-19 mRNA-based vaccine should not stop breastfeeding. In addition, any residual mRNA below the limits of detection in our assay would undergo degradation by the infant gastrointestinal system, further reducing infant exposure. Limitations of this study are the small sample size and few participants who received the mRNA-1273 vaccine. In addition, milk storage conditions may affect mRNA stability. Clinical data from larger populations are needed to better estimate the effect of these vaccines on lactation outcomes.

## Notes

Supplement.
eMethods.
