# Supplementary material for: Evaluation of Messenger RNA From COVID-19 BTN162b2 and mRNA-1273 Vaccines in Human Milk
Source: JAMA Pediatr. 2021 Jul 6;175(10):1069–71. doi: 10.1001/jamapediatrics.2021.1929 (PMC8261686; doi:10.1001/jamapediatrics.2021.1929)
Supplement: Supplement. — eMethods. [file jamapediatr-e211929-s001.pdf]

Supplemental Online Content

Golan Y, Prahl M, Cassidy A, et al. Evaluation of messenger RNA from COVID-19 BTN162b2 and mRNA-1273 vaccines in human milk. *JAMA Pediatr*. Published online July 6, 2021. doi:10.1001/jamapediatrics.2021.1929

eMethods.

This supplementary material has been provided by the authors to give readers additional information about their work.

© 2021 Golan Y et al. *JAMA Pediatrics*.

eMethods.

<u>Milk samples</u>- Fresh human milk samples were self-collected by participants 4-48h after vaccine administration. If not collected immediately for analysis, mothers were instructed to freeze milk as soon as possible after milk pumping. Samples were kept on ice until arriving in the lab for processing (up to 2 hours after milk pumping). Five ml of whole breast milk was mixed in a 1:1 ration with RNAlater (catalog number R0901; Sigma-Aldrich), aliquoted and frozen at -80°C until analyzed (Breast milk in RNAlater was treated as the frozen sample below). The remaining milk was centrifuged for 20 min at 800g at 4°C. Approximately 200ul of fat layer was collected into new Eppendorf tubes and was mixed with 600 μL of RLT buffer (RNeasy Mini Kit; Qiagen) and kept at -80°C until analyzed. Supernatant was removed and cells pellets were resuspended in 350 μL of RLT buffer (cellular fraction).

Frozen milk samples were thawed on ice and were mixed gently by pipetting up and down. 1 ml milk was mixed with RNAlater at a 1:1 ratio immediately after thawing. Samples were centrifuged for 1 min 10000g at 4°C to separate milk fat from supernatant. Milk fat was transformed to a new tube and was mixed with 600 μL RLT buffer immediately. Two hundred μL of supernatant were mixed with 600 μL RLT buffer. Samples in RLT buffer were used for RNA isolation. Fat layer samples in RLT buffer were centrifuged for 1 min 10000g at 4°C and fat was removed from samples before processing with RNA isolation protocol.

<u>BNT162b2 (Pfizer) and mRNA-1273 (Moderna) mRNA RT-qPCR</u> - RNA was isolated from samples using the RNeasy Mini Kit (Qiagen) according to manufacturer's protocol. RNA concentration was measured using nanodrop and samples that had >10ng/μl total RNA were used for RT reaction. 150-500ng RNA was transcribed into cDNA using qScript cDNA synthesis kit (Quantabio) according to the manufacturer's protocol. Primers were design to detect the vaccines mRNA, and BNT162b2 (Pfizer) and mRNA-1273 (Moderna) commercial vaccine was

© 2021 Golan Y et al. *JAMA Pediatrics*.

used to determine primers specificity and sensitivity. Forward primer: 5’AACGCCACCAACGTGGTCATC. Reverse primer: 5’GTTGTTGGCGCTGCTGTACAC.

Positive and negative controls- 30 uL of mRNA-1273 (Moderna, 200 ug/uL) vaccine was spiked into 500 uL of pre-vaccine whole milk sample from participant 1 (final concentration 12 ng/uL), this sample was diluted in 1:100 in whole milk to create the 0.12ng/μL sample. These milk samples served as positive controls to show that the vaccine mRNA is detectable in inoculated milk samples using the mRNA extraction method and RT-qPCR measurement that were used for participants samples. The vaccine-inoculated samples were mixed with RNAlater at a 1:1 ratio and treated as described above for RNA isolation from milk samples. Pre-vaccine samples collected from 4 participants served as negative controls that do not contain vaccine mRNA.

QuantaStudio 6 Flex (Applied Biosystems) instrument and SsoFast EvaGreen supermix (Bio-Rad) were used for PCR reaction: 30 second 95°C followed by 45 cycles of 5 second 95°C and 20 seconds 60°C. All samples were run in triplicate as 20 μL reactions, containing 10 ng cDNA. Ct values ≥40 were interpreted as a negative result (BDL, below detectable levels). Threshold was set based on negative controls of pre-vaccine samples and NTC. For vaccines cDNA standard curves, 100pg/μL vaccine mRNA (as cDNA) sample was used for serial dilution in 1:2 ratio, up to 0.0975 pg/μL. Two μL of these diluted samples were used in each well to create standard curves.

Calculation of sample concentration- RNA was isolated directly from mRNA-1273 and BNT162b2 vaccines using the same method described above. Vaccines cDNA in various concentrations (200-0.195 pg/well) were used to create standard curves. Log values of pg/well were plotted on X axis and Ct values on Y axis. The mRNA-1273 vaccine equation was y = -

© 2021 Golan Y et al. *JAMA Pediatrics*.

6.522x + 38.437, and *BNT162b2* vaccine equation was y = -4.8558x + 38.898. Ct values of samples were used to calculate vaccine cDNA concentrations (pg) in each well.

Effect of freeze/thaw cycle on mRNA detection- To determine the effect of freezing and thawing on the vaccine mRNA stability, we inoculated mRNA-1273 (12ng/ul as mention above) and BNT162b2 (1.2ng/ul, 120ul vaccine in 880ul milk) vaccines into pre-vaccine human milk sample. We isolated RNA from half of the sample and froze the other half overnight in a -20°C freezer, to mimic the storage conditions of frozen milk samples collected in this study. RNA was extracted from fat layer of these samples using the same method as mention above, and 10 ng/well cDNA was used for qPCR. Ct values obtained after freezing and thawing were lower compared to the samples that were not frozen, indicating that the vaccines mRNA detection of our assay was not negatively affected after a single freeze/thaw cycle.

© 2021 Golan Y et al. *JAMA Pediatrics*.
